# Improved radiological imaging of congenital aural atresia using flat-panel volume CT

**DOI:** 10.1007/s00106-024-01512-0

**Published:** 2024-11-07

**Authors:** Franz-Tassilo Müller-Graff, Jan von Düring, Johannes Voelker, Fadi Al-Tinawi, Rudolf Hagen, Tilmann Neun, Stephan Hackenberg, Kristen Rak

**Affiliations:** 1 Department of Oto-Rhino-Laryngology, Plastic, Aesthetic and Reconstructive Head and Neck Surgery and the Comprehensive Hearing Center, University of Wuerzburg, Josef-Schneider-Straße 11, 97080 Würzburg, Germany; 2 Insitute for Diagnostic and Interventional Neuroradiology, University of Wuerzburg, Würzburg, Germany

**Keywords:** OTOPLAN, Computed-tomography-based software, Atresia plate and facial nerve, Ear deformity, Middle ear malformation, OTOPLAN, Computertomographie basierte Software, Atresieplatte und Nervus facialis, Ohrmissbildung, Mittelohrfehlbildung

## Abstract

**Background:**

Precise preoperative radiological evaluation of aural atresia is of utmost importance for surgical planning. Until now, multislice computed tomography (MSCT) has been used but it cannot adequately visualize small structures such as the stapes. Flat-panel volume CT (fpVCT) with its secondary reconstructions (fpVCT_SECO_) offers a high-resolution visualization of the middle ear. New otosurgical planning software also enables detailed 3D reconstruction of the middle ear anatomy.

**Aim of the work:**

Evaluation of the use of fpVCT_SECO_ in combination with an otosurgical planning software for a more accurate diagnosis and treatment of congenital aural atresia.

**Material and methods:**

Seven patients with congenital aural atresia underwent preoperative MSCT (600 µm slice thickness) and corresponding fpVCT (466 µm slice thickness). In addition, fpVCT_*SECO*_ (99 µm slice thickness) were reconstructed. The Jahrsdoerfer and Siegert grading scores were determined and their applicability in the abovementioned imaging modalities was evaluated. In addition, the malleus incus complex was analyzed in 3D rendering.

**Results:**

Imaging with fpVCT_*SECO*_ enabled reliable visualization of the abnormalities, in particular the ossicular chain. A significant difference in the Siegert grading score was found. In addition, the malleus-incus complex could be visualized better in 3D.

**Discussion:**

The introduction of new imaging techniques and surgical planning techniques into the diagnostic concept of aural atresia facilitates the identification of malformed anatomy and enables systematic analysis. This combination can also help to more accurately classify the pathology and thus increase the safety and success of the surgical procedure.

## Abstract (de)

**Hintergrund:**

Eine präzise präoperative radiologische Evaluation von Auralatresien ist für die Operationsplanung von größter Bedeutung. Bisher wurde hierfür die Multislice-Computertomographie (MSCT) verwendet, die jedoch kleine Strukturen wie den Stapes nur unzureichend dargestellen kann. Die „flat-panel volume CT“ (fpVCT) mit ihren sekundären Rekonstruktionen (fpVCT_SECO_) bietet eine hochauflösende Darstellung des Mittelohrs. Eine neue otochirurgische Planungssoftware ermöglicht überdies die detaillierte 3‑D-Rekonstruktion der Mittelohranatomie.

**Ziel der Arbeit:**

Evaluierung des Einsatzes von fpVCT_SECO_ in Kombination mit einer otochirurgischen Planungssoftware zur genaueren Diagnose und Behandlung kongenitaler Auralatresien.

**Material und Methoden:**

Sieben Patienten mit kongenitaler Auralatresie erhielten präoperativ ein MSCT (600 µm Schichtdicke) und ein korrespondierendes fpVCT (466 µm Schichtdicke). Zusätzlich wurden fpVCT_*SECO*_ (99 µm Schichtdicke) rekonstruiert. Die Jahrsdoerfer- und Siegert-Grading-Scores wurden bestimmt und ihre Anwendbarkeit in den oben genannten bildgebenden Modalitäten bewertet. Zusätzlich wurde der Malleus-incus-Komplex im 3‑D-Rendering analysiert.

**Ergebnisse:**

Die Bildgebung mit fpVCT_*SECO*_ ermöglichte eine zuverlässige Darstellung der Anomalien, insbesondere der Gehörknöchelchenkette. Es wurde ein signifikanter Unterschied im Siegert-Grading-Score festgestellt. Zusätzlich konnte der Malleus-Incus-Komplex in 3‑D besser dargestellt werden.

**Diskussion:**

Die Einführung neuer bildgebender Verfahren und chirurgischer Planungstechniken in das diagnostische Konzept der Auralatresie erleichtert die Identifizierung der fehlgebildeten Anatomie und ermöglicht eine systematische Analyse. Diese Kombination kann auch dazu beitragen, die Pathologie genauer zu klassifizieren und damit die Sicherheit und den Erfolg des chirurgischen Eingriffs zu erhöhen.

Congenital aural atresias are rare congenital malformations that are associated with functional and cosmetic limitations for those affected. Accurate radiological imaging of these pathologies is often not possible using conventional imaging techniques due to technical limitations. New radiological techniques and computer-aided software are promising developments, especially for children, where optimal treatment should be guaranteed with a view to possible surgical hearing reconstruction.

Congenital aural atresia is characterized by hypoplasia or aplasia of the external auditory canal in conjunction with a dysmorphic pinna. Furthermore, the middle ear and in particular the ossicles and occasionally also the inner ear are malformed [19]. The estimated incidence of aural atresia is about 1 in 11,000–15,000 newborns [17]. Aural atresia can occur in an isolated form or as part of a syndrome [4, 23].

Imaging techniques are essential during the clinical diagnosis for assessing the severity of the aural atresia and for the preoperative evaluation of a possible surgical intervention. Currently, computed tomography (CT) is recommended as the diagnostic imaging method of choice, as it helps the surgeon to predict the anatomy of the middle ear and provides a prognostic basis for surgical intervention [23].

Jahrsdoerfer et al. described a radiological grading score for a more precise classification of aural atresia from a surgical point of view, which is frequently used in clinical practice [8]. This score can help in the selection of patients who have a realistic chance of a classic surgical restoration of hearing. Siegert et al. extended and supplemented this classification with a semi-quantitative system for the assessment of surgical indications [22]. With conventional multislice computed tomography (MSCT), however, small structures such as the stapes, which are very important for classification and also for the success of the operation, cannot be adequately depicted. Flat-panel volume computed tomography (fpVCT) is a new CT technique that enables visualization with very high spatial resolution. In several studies, a significantly better visualization of the fine bony structures of the middle ear and the petrous temporal bone was achieved [9] compared with MSCT, and a reduction in the effective dose by approx. 40% was possible compared with 64-slice MSCT [6] and 128-slice MSCT [16].

In addition, fpVCT offers the possibility of even improving image quality and spatial resolution through its secondary reconstructions (fpVCT_SECO_) without the need for further radiation exposure [15]. As far as we are aware, this technique has not been the subject of investigations for congenital aural atresia. This involves a reduction in the volume of interest (VOI) while the matrix remains the same, which reduces the slice thickness and increases the resolution within the VOI. It is helpful for further image processing that the fpVCT technique generates isotropic voxels with the same edge length. By comparison, MSCT generates anisotropic voxels with unequal edge lengths. The term “secondary reconstruction” was coined by the first author of this method to emphasize the fact that the changes in the VOI and thus the slice thickness occur after the reconstruction of the slice images from the primary dataset [15].

The newly developed otological planning software OTOPLAN® (CAScination, Bern, Switzerland, in collaboration with MED-EL, Innsbruck, Austria) is a tablet-based software designed for the preoperative planning of otological procedures [7, 12, 13]. One of the main functions is the visualization of the individual middle ear structures, which enables the image data to be displayed in the three body axes and a 3D visualization through automated segmentation, making it possible to compare the different imaging modalities [3].

These digital tools are a great help to surgeons in preoperative planning, especially in the case of young patients who benefit from maximum hearing gain due to their development. To date, only malformations of the inner ear have been analyzed using this software [2] but no cases of aural atresia.

The aim of this study was to investigate whether fpVCT (466 µm slice thickness) and in particular its secondary reconstructions (fpVCT_SECO_—99 µm slice thickness) facilitate the radiological diagnosis of congenital aural atresia in comparison with conventional MSCT (600 µm slice thickness). To answer this question, (1) we investigated whether the radiological grading scores according to Jahrsdoerfer and Siegert differ when using the different imaging modalities. Furthermore, (2) the suitability of fpVCT_SECO_ in combination with the new otological planning software for the diagnosis of congenital atresia was evaluated. Finally, (3) the 3D visualization of the malleus–incus complex in the otological planning software was investigated.

## Methods

### Participants

In this retrospective study, 7 petrous bone scans of patients with congenital aural atresia (6 unilateral, 1 bilateral), which had been performed as part of the preoperative diagnosis, were analyzed. Only patients who had undergone both an MSCT and an fpVCT examination were included in the study. In our institution, we routinely use fpVCT with secondary reconstructions (fpVCT_SECO_) for all perioperative planning of hearing implants due to its high resolution. If a previous temporal bone CT, usually performed at an external facility, was available, it was utilized for the retrospective data analysis in this study. Secondary reconstructions (fpVCT_SECO_) were created from the data of the fpVCT scans. The age of the patients at the time of imaging ranged from 15 to 59 years (mean: 35 years, median: 31 years). Four ears with aural atresia were left-sided, three were right-sided. The middle ear structures were visualized using the otological planning software OTOPLAN® (CAScination, Bern, Switzerland, in collaboration with MED-EL, Innsbruck, Austria). Scans of the unaffected contralateral side of the 6 patients with unilateral aural atresia were used as controls.

This retrospective, anonymized study was conducted in accordance with local guidelines and the principles of the Declaration of Helsinki and Good Clinical Practice and was approved by the local ethics committee (2021050601).

### Imaging

The MSCT datasets were acquired on the MSCT scanners SOMATOM Definition AS+ (Siemens), Brilliance 40 (Philips), Alexion (Toshiba), and Aquilion Prime (Toshiba) with an average slice thickness of 600 µm. The median values of the tube parameters were the following: tube current = 75 mA; tube voltage = 114 kV. The median examination time was 3.7 s, if indicated in the external imaging.

The fpVCT scans were acquired using an angiography device (Axiom Artis, Siemens Healthcare AG, Erlangen, Germany) with commercially available software (Syngo DynaCT, Siemens). The datasets were acquired with the following median parameters: 20 s DCT head protocol; tube current = 21 mA; tube voltage = 109 kV; rotation angle = 200°; pulse length = 3.5 ms; frame angulation step = 0.5°/image; mean slice thickness = 466 µm. From these datasets, the fpVCT_SECO_ was developed according to the findings of Pearl et al. with the following settings: 512 × 512 section matrix; HU kernel types; sharp image features; median slice thickness = 99 µm [15].

### Evaluation of different imaging modalities for the examination of aural atresia

To assess the congenital aural atresia, the datasets were converted into the DICOM standard via the hospital’s PACS network. They were then transferred anonymously to the otological planning software and the Jahrsdoerfer and Siegert grading scores were determined. All structures identified for the determination of these scores are listed in Table 1*.*

**Table 1:** Classification of the grading scores for aural atresia

Structure	Jahrsdoerfer score	Siegert score
Configuration	Present/not present (points)	Normal/slightly dysplastic/severely dysplastic (points)
Stapes	2/0	4/2/0
Malleus–incus complex	1/0	2/1/0
Incudostapedial joint	1/0	–
Facial nerve	1/0	4/2/0
Round window	1/0	4/0
Oval window	1/0	4/0
Mastoid pneumatization	1/0	2/1/0
External ear canal	1/0	2/1/0
Middle ear size and aeration	1/0	4/2/0
Course of arteries and veins	–	2/1/0
Maximum score	*10*	*28*

The various anatomical substructures of the ossicles, whether normal or malformed, were then anatomically examined in more detail using a modified scale and rating list, as described by Majdani et al. [9], which resulted in an extended score, the so-called ossicle score. Here, the differentiation from the surrounding tissue was determined using a numerical scale from 0 to 3: 0 = The structure was not recognized, 1 = the structure could not be easily differentiated from the surrounding structures, 2 = the structure was moderately differentiated, and 3 = the structure was well differentiated from the surrounding tissue. Based on this rating scale, the identification of the different structures was calculated as a percentage.

The visualization of the malleus–incus complex with the different imaging modalities was performed with the automatic segmentation tool guided by the planning software. Based on the user’s individual selection of a single point around the incudomalleolar joint, a combined segment for the complex was created.

The Jahrsdoerfer and Siegert scores were compared between the three imaging modalities for all participants. The analysis was performed in a series of tests by two different examiners who were highly experienced in the assessment of temporal bone imaging (senior ENT consultant [KR] and ENT specialist [FTMG], both with a focus on otology, supervised by a neuroradiology specialist [TN]). Patient data were anonymized. The order of analysis was randomized in three ways: randomization of imaging modality, randomization of petrous bone, and randomization of score structures.

### Statistical analysis

Prior to the parametric analyses, the normal distribution of all data series was confirmed through the Kolmogorov–Smirnov test. One-way repeated-measures analysis of variance (ANOVA) was used to assess the differences in absolute mean grading scores across the three modalities and settings. For multiple comparisons, the Tukey test for multiple comparisons was used. Differences with a *p *value of less than 0.05 were considered statistically significant.

Statistical analyses and the creation of diagrams were carried out using GraphPad Prism (version 8.4.0, San Diego, CA, USA) and IBM SPSS Statistics (version 25.0.0.0; IBM Corporation, Armonk, NY, USA). The data are presented in bar charts.

## Results

### Middle ear structures

In order to assess the degree of middle ear pathology in aural atresia, a radiological diagnostic examination of the petrous temporal bone was performed for all patients. Structures that contribute to both the Jahrsdoerfer and Siegert scores were visualized from the image data. These include the malformed ossicles, deviations in the course of the facial nerve, configurations of the oval and round window, and the pneumatization of the mastoid cells and the auditory canals. All structures could be visualized more precisely with fpVCT_SECO_ compared to MSCT in terms of their anatomy and integrity. Selected structures are described in more detail below.

#### Stapes.

**Fig. 1:**
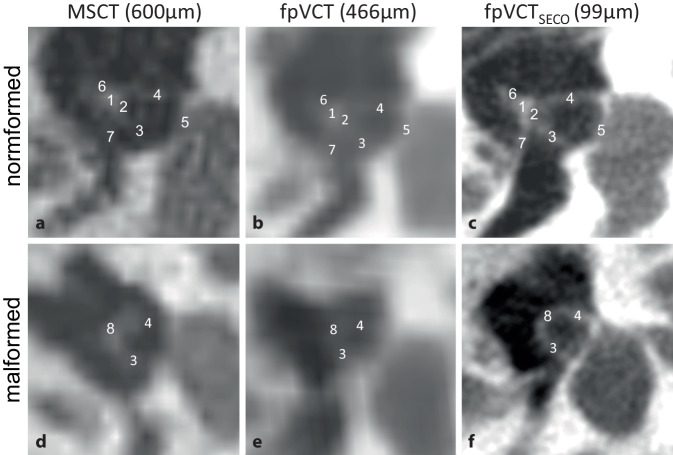
View of the stapes in different imaging modalities and settings. Representative image of a non-malformed stapes using **a** MSCT (600 µm slice thickness), **b** fpVCT (466 µm slice thickness), and **c** fpVCT_SECO_ (99 µm slice thickness). **d**–**f** Demonstration of the stapes view in malformations. The following stapes substructures were identified: *1* caput stapedis, *2* collum stapedis, *3* crus anterius stapedis, *4* crus posterius stapedis, *5* basis stapedis, *6* articulatio incudostapedialis, *7* m.stapedius, *8* malformed stapes. *MSCT* multislice computed tomography, *fpVCT* flat-panel volume computed tomography, *fpVCT*_*SECO*_ secondary reconstructions of fpVCT

The stapes could be visualized very precisely with fpVCT_SECO_. Instead of only the schematic visualization with MSCT, fpVCT_SECO_ also showed details of various substructures such as the caput and collum. In addition, both crura, if present, were visualized in their entirety, and in many cases the footplate (basis stapedis) was also recognized. In excellent imaging examples, the stapedius muscle was also identified. As an important connection to the malleus–incus complex, the incudostapedial joint could also be visualized more clearly with the fpVCT_SECO_. An example of a normal and a malformed stapes in the different imaging modalities is shown in Fig. 1.

#### Malleus incus complex.

**Fig. 2:**
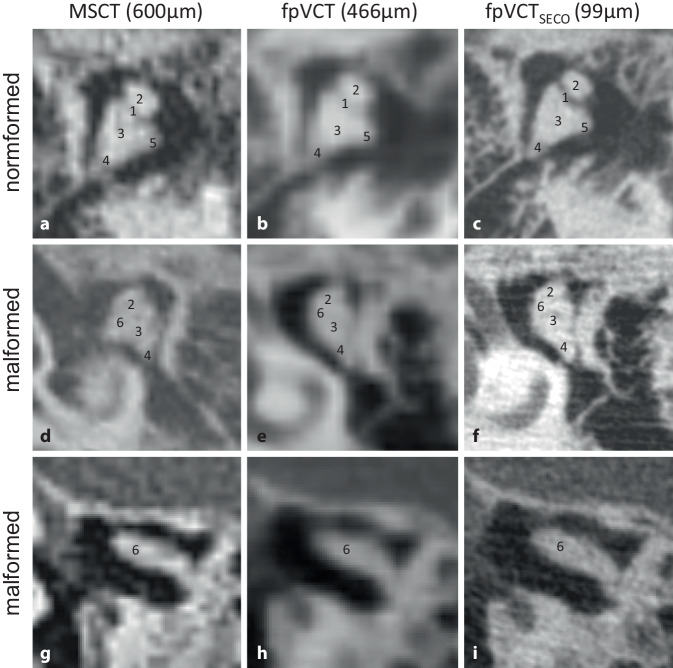
Visualization of the malleus–incus complex with different imaging modalities and settings. A representative image using **a** MSCT (600 µm slice thickness), **b** fpVCT (466 µm slice thickness), and **c** fpVCT_SECO_ (99 µm slice thickness). **d**–**f** Respective images demonstrating the malleus–incus complex with mild malformation and **g**–**i** with severe malformation. The following substructures of the malleus–incus complex were identified: *1* articulatio incudomalleolaris, *2* caput mallei, *3* corpus incudis, *4* crus breve incudis, *5* crus longum incudis, *6* fused art. incudomalleolaris. *MSCT* multislice computed tomography, *fpVCT* flat-panel volume computed tomography, *fpVCT*_*SECO*_ secondary reconstructions of fpVCT

An example of a regular, slightly malformed and severely malformed complex in the different imaging modalities is shown in Fig. 2. Although the incudomalleolar joint was delineated in some MSCT datasets, reliable identification was only possible with fpVCT_SECO_. In low-resolution imaging, it was therefore often not possible to clearly distinguish whether the joint was fused or only poorly visualized radiologically.

The results of the ossicle score are shown in Table 2*. *The stapes structures as well as the structures of the incudostapedial joint and the malleus–incus complex were identified with the highest percentages using fpVCT_SECO_.

**Table 2:** Ossicle score results (%) for the identification of anatomical structures of the auditory ossicles, if present, in patients with aural atresia

	MSCT	fpVCT	fpVCT_SECO_
Patients	7	7	7
Number of the rater	2	2	2
**–**	**Identification (%)**	**Identification (%)**	**Identification (%)**
*Stapes*			
Head	45.83	54.17	83.33
Collum	20.83	37.50	75.00
Crus anterior	30.00	36.67	70.00
Crus posterior	13.33	40.00	70.00
Footplate	26.67	60.00	76.67
Mean	*27.33*	*45.67*	*75.00*
*Incus/stapes*			
Incudostapedial joint	*25.00*	*33.33*	*83.33*
*Malleus/incus*			
Malleus–incus complex	86.67	90.00	100.00
Corpus incudis	86.67	80.00	100.00
Caput mallei	80.00	80.00	100.00
Incudomalleolar joint	37.50	44.44	87.50
Crus breve incudis	37.33	73.33	100.00
Crus longum incudis	58.33	70.37	100.00
Mean	*70.42*	*73.03*	*97.92*

#### Facial nerve.

**Fig. 3:**
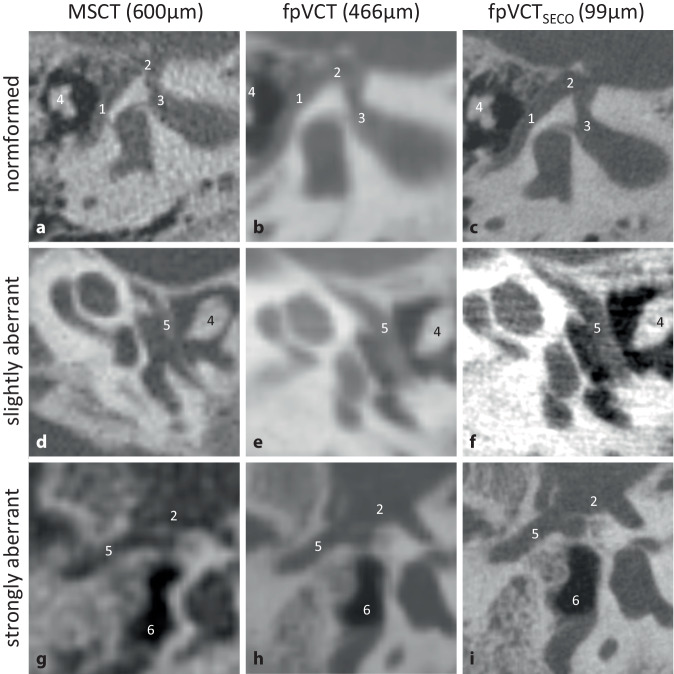
Visualization of the course of the facial nerve. A representative image with **a** MSCT (600 µm slice thickness), **b** fpVCT (466 µm slice thickness), and **c** fpVCT_SECO_ (99 µm slice thickness). Respective images **d**–**f** demonstrating a slightly aberrant course of the facial nerve through the tympanic cavity and **g**–**i** a strongly anomalous course of the facial nerve. *1* Canalis nervi facialis, *2* ganglion geniculatum, *3* meatus acusticus internus, *4* malleus incus complex, *5* dysmorphic course of the facial nerve, *6* dysmorphic reduced tympanic cavity. *MSCT* multislice computed tomography, *fpVCT* flat-panel volume computed tomography, *fpVCT*_*SECO*_ secondary reconstructions of fpVCT

Of the three imaging modalities examined, FpVCT_SECO_ provided the most accurate visualization of the facial nerve, which is crucial for the surgical procedure. In particular, a better delineation was recognizable when the nerve deviated through the tympanic cavity. Representative images of a normal as well as a slightly and strongly deviated course of the facial nerve are shown in Fig. 3.

#### Oval and round window.

The exclusion of ossification of the cochlear windows was generally possible with all three imaging modalities. However, if the low-resolution imaging showed an unfavorable sectional plane, it could give the impression that a window was ossified, which proved to be open at the higher resolution.

#### Mastoid pneumatization and external auditory canal.

**Fig. 4:**
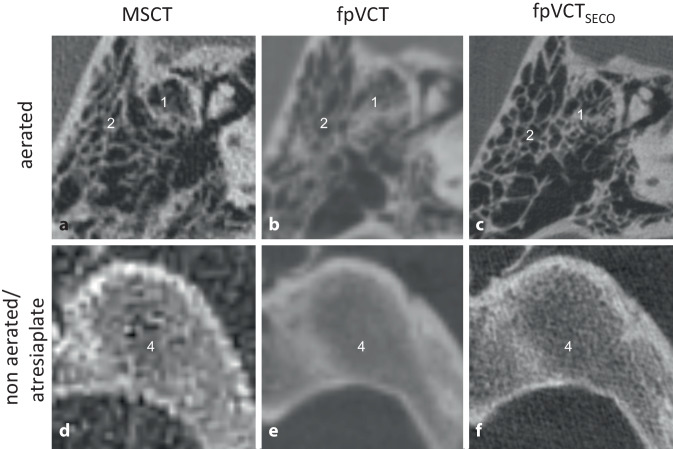
Visualization of the aeration of the mastoid cells. A representative image with **a** MSCT (600 µm slice thickness), **b** fpVCT (466 µm slice thickness), and **c** fpVCT_SECO_ (99 µm slice thickness). **d**–**f** Images displaying a non-aerated/atresized plate. *1* Ventilated mastoid cells, *2* bony atresia of the external auditory canal, *4* mastoid atresia plate. *MSCT* multislice computed tomography, *fpVCT* flat-panel volume computed tomography, *fpVCT*_*SECO*_ secondary reconstructions of fpVCT

Absence of the external auditory canal was detected with all imaging modalities. However, individual mastoid cells were much more difficult to delineate with low-resolution imaging. Accurate visualization, especially when differentiating between a completely atretic bone and the remaining aerated mastoid cells, was only possible with fpVCT_SECO_ (Fig. 4).

## 3D visualization of the auditory ossicles

**Fig. 5:**
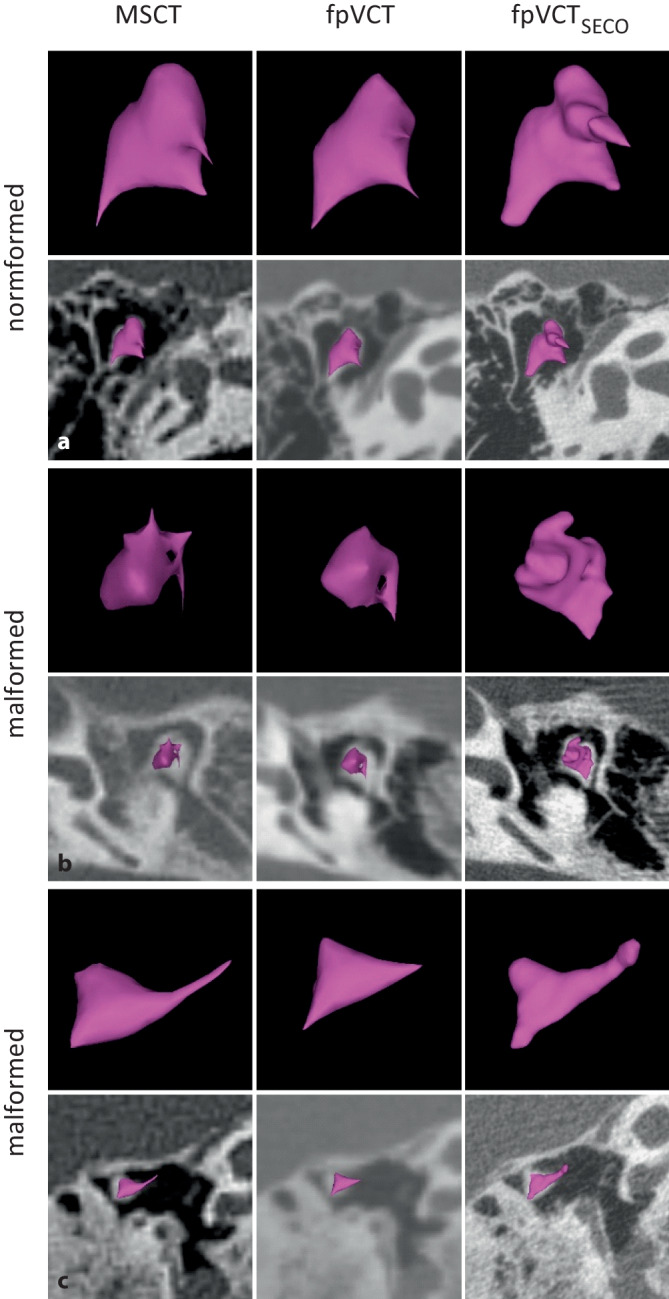
3D visualization of the malleus–incus complex in the OTOPLAN planning software® using MSCT (600 µm slice thickness), fpVCT (466 µm slice thickness), and fpVCT_SECO_ (99 µm slice thickness). The complexes are each shown in isolation and with an overlaid axial plane. A normally configured complex **a**, a moderately malformed complex **b**, and a severely malformed complex **c** are shown. *MSCT* multislice computed tomography, *fpVCT* flat-panel volume computed tomography, *fpVCT*_*SECO*_ secondary reconstructions of fpVCT

To compare the 3D visualization of the different imaging modalities, the malleus–incus complex was segmented using the automatic tool of the surgical planning software. As Fig. 5 shows, the visualization of the complex with fpVCT_SECO_ was much more detailed and anatomically correctly segmented compared with MSCT; MSCT did not depict the malformed complexes satisfactorily.

### Comparison of grading scores

**Fig. 6:**
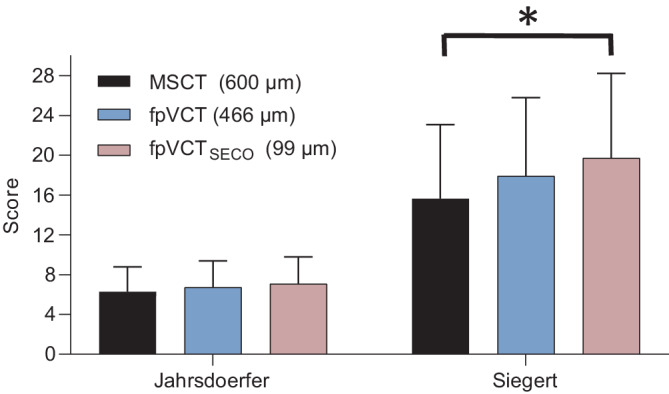
Analysis of CT grading scores for aural atresia in different radiological settings and modalities. The Jahrsdoerfer score is shown *on the left* and the Siegert score *on the right*. The number of temporal bones examined was *n* = 7. The grading scores were assessed by two experts. Differences between the indicated cohorts are shown as significance, ^*^p = 0.05. *MSCT* multislice computed tomography, *fpVCT* flat-panel volume computed tomography, *fpVCT*_*SECO*_ secondary reconstructions of fpVCT

The values for the three imaging modalities are shown in Fig. 6*. *The mean value of the Jahrsdoerfer grading score (reference range: 0–10 points) was 6.3 (SD: 3.4) for MSCT, 6.7 (SD: 3.2) for fpVCT, and 7.1 (SD: 2.9) for fpVCT_SECO_. The comparison of the mean values revealed no significant differences. There were also no significant differences between the groups. When analyzing the Siegert score, the mean value (reference range: 0–28 points) was 16.2 for MSCT (SD: 11.9), 18.6 for fpVCT (SD: 10.7), and 20.4 (SD: 9.4) for fpVCT_SECO_. The comparison of the mean values revealed a significant difference (*p* = 0.0125). The difference between the evaluation using fpVCT_SECO_ and MSCT was also significant (mean 4.1; *p* = 0.0125).

## Discussion

In the case of congenital aural atresia, a major challenge is the adequate preoperative assessment of the pathology, which is crucial for the correct decision on the most effective type of surgery and the best hearing improvement. This generally requires a solid radiological diagnosis, especially in young patients. The data presented here show that preoperative imaging with secondary reconstructions of flat-panel volume CT (fpVCT_SECO_) facilitates the systematic analysis of individual anatomy, including middle ear abnormalities. In addition, 3D visualization with the otological planning software can improve anatomical accuracy.

Conventionally used MSCT imaging (600 µm slice thickness) was compared with fpVCT imaging (466 µm slice thickness) and its secondary reconstructions (fpVCT_SECO_; 99 µm slice thickness). The respective structures were analyzed to determine the Jahrsdoerfer and Siegert grading scores and a new, self-developed score (“ossicle score”) was calculated. The identification of the ossicles is of utmost importance in the radiological assessment of aural atresia. The substructures of the stapes, the incudostapedial joint, and the malleus–incus complex could be identified most accurately with the fpVCT_SECO_, as shown in Table 2. In particular, it was found that the stapes was only recognizable in its entirety and delineated from the surrounding tissue with the fpVCT_SECO_ (Fig. 1) but not with the fpVCT and MSCT, where only rudimentary fragments of the stapes were partially visible. Differentiation between normal and malformed stapes was often not possible in these two modalities. This is important because the stapes, with two points in the Jahrsdoerfer and four points in the Siegert score, is the most important structure in the Jahrsdoerfer and one of the most important in the Siegert scoring systems. The improved imaging also gains clinical relevance, as the stapes is a preferred localization for the placement of the so-called floating mass transducer (FMT) of the vibrant sound bridge (VSB) in aural atresia, and the presence of a stapes correlates with the initial postoperative threshold for aided speech reception in patients with aural atresia [11]. Therefore, a present, malformed, or absent stapes may provide some guidance for planning the position of the FMT.

In addition, the planning of an otosurgical procedure requires precise and careful preoperative assessment of other anatomical structures [1], which is improved by fpVCT_SECO_ (Figs. 2, 3, and 4). An abnormal course of the facial nerve does not necessarily preclude the possibility of surgery, and good audiological results can still be achieved. However, the risk of complications increases in such situations [10, 23]. The identification and delineation of the facial nerve have been improved with fpVCT_SECO_, which could increase the safety of the planned surgical procedure. The use of fpVCT_SECO_ can also improve the correct indication and planning of the procedure and help to predict contraindications such as obstruction of the oval window by a deviated facial nerve [23].

The data from the different imaging modalities were additionally visualized using the automatic segmentation tool of the otologic planning software OTOPLAN® to display the malleus–incus complex in 3D (Fig. 5). The imaging properties of this structure, especially in malformed configuration, were reliably visualized by fpVCT_SECO_. The use of MSCT and fpVCT, on the other hand, tended to result in cuboidal objects that appeared glued together (Fig. 5), which did not correspond to reality. This is due to the threshold-based software method. Low-resolution imaging methods have wider widths at the thresholds and, especially at the edges, a lower contrast to the surrounding tissue. As a result, the segmentation of low-resolution images cannot adequately depict structures in 3D. Consequently, accurate 3D visualization is not yet possible. Particularly at the outer boundaries of the complex, certain areas are not yet captured despite the improved imaging with fpVCT_SECO_. However, in the future, fpVCT_SECO_ could offer the possibility of automatically segmenting the stapes in otological software solutions, which is currently not possible due to the relatively low resolution in clinical imaging. The combination of fpVCT_SECO_ and the advanced software could help to classify otological pathologies more precisely and thus also increase the safety and success of the therapy.

The patients with aural atresia included in this study had a wide range of severity, with Jahrsdoerfer scores of 3–9 (reference value: 0–10) and Siegert scores of 5–26 (reference value: 0–28). Calculation of the grading scores revealed a significant difference in Siegert score between the higher scores obtained with fpVCT_SECO_ compared with the scores that assessed the same petrous temporal bone with MSCT and fpVCT (Fig. 6). Assuming that fpVCT_SECO_ most accurately reflects the actual anatomy in the imaging procedures examined, one could speculate that the indication for the type of surgery proposed could be reconsidered. This is especially true for severe cases of aural atresia. In some of these cases, a higher score would be achieved, which could potentially favor surgical intervention, as recent publications suggest that a Jahrsdoerfer score over 3 can already lead to an excellent audiological outcome [5]. In other words: In borderline cases, it would be possible for a patient not to be considered a good candidate for atresia surgery or middle ear implantation based on MSCT, but they may be considered a good candidate on the basis of fpVCT_SECO_ imaging.

Overall, CT imaging is also justified for pediatric patients in order to clarify and consider the following aspects:

(i) The exact diagnosis with determination of the type and extent of the aural atresia
(ii) The assessment of potential concomitant abnormalities in the head region
(iii) Developing a sound treatment plan, possibly including surgical intervention, identifying in advance the best approach to minimize potential risks and challenges
(iv) Performing follow-up visits to monitor changes over time and adjust the course of treatment as needed

However, it should be noted that the population studied here was mainly adolescents and young adults, in whom CT can usually be performed in the awake state without any problems. By contrast, it seems reasonable to perform CT in young children under short anesthesia or sedation to minimize the risk of motion artifacts. However, as preoperative hearing assessment with, for example, brainstem evoked response audiometry (BERA) is always mandatory for patients of this young age, both hearing assessment and imaging could be performed under common short anesthesia/sedation in such cases and thus be justified. Nevertheless, it should be carefully considered on a case-by-case basis whether diagnostic examinations with ionizing radiation are really necessary in childhood or can be performed at a later date. This is especially true if surgery is planned for a later date, such as in adolescence or early adulthood. Ideally, therefore, young patients with aural atresia should only undergo CT imaging if the parents have a clear understanding of the pathology and a resulting interest and desire for possible surgery.

### Limitations of the study

Since patients with aural atresia are often referred for external imaging, it should be mentioned that the MSCT imaging data were acquired with different devices but with similar slice thickness. This could have influenced the evaluation. In addition, there was sometimes a temporal difference between the MSCT and fpVCT scans, which may have led to a bias, particularly with regard to the ventilation of the mastoid cells.

For radiation hygiene reasons, care is taken to perform only one CT scan if possible, especially in young patients, which limits the sample size (seven petrous bones from six patients), as often not all three imaging modalities of the same temporal bone are available for comparison. However, previous radiologic studies that examined cochlear duct length in the cochlea, for example, have shown that this number of patients is adequate to achieve sufficient statistical significance [14, 18, 20, 21]. Nevertheless, the results show that higher-resolution imaging depicts the anatomy more accurately and provides more reliable indications, especially in the case of pronounced malformation.

## Practical conclusion

- The study shows that imaging with higher resolution, such as flat-panel volume computed tomography with secondary reconstructions (fpVCT_SECO_) with 99 µm slice thickness, can depict congenital aural atresia more accurately.
- This is particularly important in clinical practice for planning hearing-improving surgery.
- Especially in pediatric patients who require optimal care, this offers the best assessment of the pathology with acceptable radiation exposure. It is therefore recommended to also use fpVCT imaging with secondary reconstructions for aural atresia.

## Data Availability

The authors confirm that all data generated or analyzed during this study are included in this article.

## Declarations

This retrospective, anonymized study was conducted in accordance with local guidelines and the principles of the Declaration of Helsinki and Good Clinical Practice and was approved by the local ethics committee (2021050601).

The supplement containing this article is not sponsored by industry.
